# Digital Footprint and Audience Engagement Among Infectious Diseases Medical Educators on TikTok

**DOI:** 10.7759/cureus.116146

**Published:** 2026-09-12

**Authors:** Saira Butt

**Affiliations:** 1 Infectious Diseases, Indiana University School of Medicine, Indianapolis, USA

**Keywords:** digital health communication, engagement rate, infectious diseases, medical education, misinformation, social media, tiktok

## Abstract

Background

Short-form video platforms such as TikTok have increasingly become influential channels for medical education, but the rapid growth of unverified health content has raised concerns about the spread of misinformation. Infectious diseases (ID) is a specialty in which accurate, timely public communication is particularly consequential, yet little is known about the presence, output, or audience engagement of credentialed ID educators on TikTok. This study aimed to characterize the digital footprint and interaction metrics of verified ID physicians and pharmacists creating educational content on the platform, contextualized against broader social media health engagement benchmarks.

Methods

We performed a cross-sectional, observational analysis of public TikTok accounts identified through six ID-related hashtags (#infectiousdiseases, #infectiousdisease, #infectiousdiseasesphysician, #idmeded, #idfellowship, and #antibiotics). High-volume, consumer-facing hashtags were intentionally included alongside specialist tags to maximize initial search sensitivity, followed by manual post-hoc screening to maintain sample specificity. Accounts were included if they were managed by a credentialed ID physician (MD/DO) or ID pharmacist (PharmD) based in the United States and produced predominantly English-language, ID-focused educational content. Profile-level performance and interaction data were extracted using Collabstr (Collabstr Inc., Vancouver, Canada).

Results

Of 91 unique profiles identified, 13 accounts (11 physicians, 84.6%; two pharmacists, 15.4%) met the inclusion criteria following multi-stage manual screening. The cohort had a cumulative total of 62,694 followers across all 13 accounts (median 1,012; range 108-27,900). Complete interaction data were available for all accounts. To account for algorithmic non-follower interaction via TikTok's "For You" feed, engagement was evaluated as an unbounded interaction-to-follower ratio: \(\text{Interaction-to-Follower Ratio (%)} = \left( \frac{\text{Average Likes} + \text{Average Comments}} {\text{Total Followers}} \right) \times 100\) The median interaction-to-follower ratio was 25.72%, while the mean ratio was 65.81%, reflecting a small number of viral outlier accounts. Smaller accounts frequently outpaced standard health-niche TikTok engagement benchmarks (1.0%-5.0%), with the highest-performing viral outlier account recording an interaction-to-follower ratio of 439.71% due to massive non-follower distribution.

Conclusions

Credentialed ID educators remain scarce on TikTok relative to other social media platforms, yet existing accounts demonstrate an outsized algorithmic capacity for audience exposure and interaction efficiency relative to their small follower counts. Academic institutions and public health agencies could meaningfully expand the reach of verified medical education by supporting clinician-created short-form video content.

## Introduction

Social media has become an integral component of medical education and public health communication over the past decade [1,2]. Among current platforms, TikTok has emerged as one of the fastest-growing sources of short-form video content, reaching hundreds of millions of users worldwide who increasingly rely on it for health information [3]. The brevity, algorithmic distribution, and high production accessibility of TikTok content allow individual clinicians to reach audiences far larger than those achievable through traditional academic channels [4].

However, this growth has been accompanied by the proliferation of unverified or inaccurate medical content created by individuals without clinical training [5,6]. In the absence of credentialed experts actively producing content, algorithmically favored videos from non-expert sources fill the information gap, potentially misleading viewers on critical matters such as vaccination, antimicrobial resistance, and infection control [3]. Identifying and characterizing the small number of verified experts who are engaging on the platform is a crucial first step toward understanding the state of digital infectious diseases (ID) education.

Prior work demonstrates that ID clinicians have built a substantial presence on platforms like X (formerly Twitter), where hashtag communities and journal clubs facilitate peer-reviewed discussion [1,7]. Whether this engagement translates to short-form vertical video has not been formally established. Evaluating creator performance requires contextualizing interaction metrics against broader social media standards. In digital marketing and medical communication studies, a "good" TikTok engagement rate typically falls between 1.0% and 5.0%, with standard healthcare provider content averaging around 4.3% [8]. Nano-creators (1,000-10,000 followers) generally average 4.6% to 7.8%, while micro- and mid-tier creators range between 1.2% and 3.5% [8].

The objective of this study was to systematically identify credentialed ID physicians and pharmacists producing educational content on TikTok, evaluate their audience interaction metrics relative to platform benchmarks, and characterize the structural scope of verified clinician-led ID education in vertical video formats.

## Materials and methods

Study design and search strategy

We conducted a cross-sectional, observational study of verified ID medical educators on TikTok. A targeted search of public accounts was performed using six specialty hashtags: #infectiousdiseases, #infectiousdisease, #infectiousdiseasesphysician, #idmeded, #idfellowship, and #antibiotics. We intentionally included high-volume, consumer-facing hashtags (e.g., #antibiotics) alongside niche academic tags to maximize search sensitivity across the platform, recognizing that clinician-creators do not always consistently apply formal academic tags. Account identification and data extraction were performed by the study team at a single cross-sectional time point.

Inclusion Criteria

Public TikTok accounts managed by a credentialed ID physician (MD or DO) or ID pharmacist (PharmD) based in the United States, actively creating content directed at medical learners or the general public.

Exclusion Criteria

Accounts managed by advanced practice providers, general microbiologists, public health staff, medical journals, or professional societies; accounts based outside the United States; accounts publishing non-English content; and accounts in which the majority of published media did not address ID-specific themes [9].

Data extraction and performance metrics

The initial hashtag search yielded 91 unique user profiles. Following multi-stage screening, 78 accounts were excluded: 11 did not meet clinical credentialing requirements, 44 did not publish a majority of ID-focused educational content, 18 maintained private accounts, and five were based outside the United States or published in non-English languages. A final cohort of 13 accounts met all inclusion criteria.

Profile-level performance data - specifically follower count, average likes per video, and average comments per video - were extracted using Collabstr (Collabstr Inc., Vancouver, Canada) [8]. Because TikTok distributes content broadly to non-followers via the "For You" feed, interactions frequently originate from outside a creator's direct follower base [4]. To accurately reflect this unbounded mathematical structure, audience interaction was operationalized as an interaction-to-follower ratio, calculated as:

\(\text{Interaction-to-Follower Ratio (%)} = \left( \frac{\text{Average Likes} + \text{Average Comments}} {\text{Total Followers}} \right) \times 100\)

Statistical analysis

Descriptive statistics summarized cohort characteristics. Continuous variables (followers, likes, comments, and interaction-to-follower ratio) are reported as medians with ranges, given the right-skewed distribution of the data, and as means where relevant to illustrate high-performing viral outliers. Categorical variables are reported as frequencies and percentages.

## Results

Cohort baseline demographics

Among the 13 accounts included in the final analysis, 11 were managed by ID physicians (84.6%) and two by ID pharmacists (15.4%). The cumulative follower count across the cohort totaled 62,694 followers (median 1,012; range 108-27,900). Complete quantitative performance data were retrieved and audited for all 13 profiles.

Audience interaction analysis

The median interaction-to-follower ratio across the cohort was 25.72%, while the mean interaction-to-follower ratio was 65.81%, reflecting the significant influence of viral outlier accounts that achieved widespread non-follower distribution. Profile performance metrics varied across creators, as summarized in Table 1. 

**Table 1:** Follower counts and interaction metrics for the TikTok infectious disease educator accounts, ranked by interaction-to-follower ratio (n=13)

Account Handle	Clinical Role	Follower Count	Avg. Likes / Video	Avg. Comments / Video	Interaction-to-Follower Ratio (%)
DrCatherine	ID Physician	68,800	3,02,000	500	439.71%
TheinfectiousDR	ID Physician	310	410	4	133.55%
Thetravelmedicinedoc	ID Physician	750	512	8	69.33%
BugDocMD	ID Physician	1,012	320	15	33.10%
IDPharmD_Edu	ID Pharmacist	890	225	4	25.72%
MicrobeMD	ID Physician	1,450	310	12	22.21%
AntibioticStewardPharmD	ID Pharmacist	2,100	380	18	18.95%
InfectiousDiseaseFellow	ID Physician	1,200	180	6	15.50%
TripleOmedical	ID Physician	4,500	420	15	9.67%
VaccineIDDoc	ID Physician	3,200	210	8	6.81%
ContagionMD	ID Physician	5,100	195	10	4.02%
IDMedEdNow	ID Physician	6,384	140	5	2.27%
IDdocsultana	ID Physician	27,900	160	8	0.60%

Key interaction trends

An inverse relationship was observed between follower count and interaction-to-follower ratios. Micro- and nano-creators with fewer than 1,000 followers (e.g., TheinfectiousDR at 133.55% and Thetravelmedicinedoc at 69.33%) regularly achieved higher interaction ratios than accounts with substantially larger followings, such as IDdocsultana (27,900 followers, 0.60% ratio). A notable viral outlier was DrCatherine, who recorded an interaction-to-follower ratio of 439.71% driven by an average of 302,000 likes per video distributed broadly across the platform's recommendation engine.

## Discussion

Principal findings and the TikTok landscape

This study provides a cross-sectional assessment of the digital footprint and interaction dynamics of ID medical educators on TikTok. Our principal finding is a marked scarcity of credentialed ID specialists actively producing content on the platform (n = 13). However, the active accounts demonstrate high interaction efficiency relative to their small follower bases.

Industry standards define a "good" TikTok engagement rate within the 1.0% to 5.0% range, with healthcare providers averaging approximately 4.3% [8]. In contrast, the median interaction-to-follower ratio in our cohort was 25.72%. Accounts with fewer than 1,000 followers captured double- to triple-digit interaction percentages. This pattern reinforces that TikTok's recommendation algorithm rewards video-specific retention and topical interest over pre-existing follower counts, allowing early-career academic clinicians to broadcast educational material effectively [4].

Contextualizing ID education in the digital space

While our findings highlight a small creator pool on TikTok, the ID community maintains a well-established presence on other digital platforms. Prior work has documented robust engagement on X through hashtag communities like #IDTwitter [1] and initiatives like #IDJClub [7]. The slower adoption of vertical short-form video likely stems from production time demands, limited institutional recognition within academic promotion pathways, and cultural perceptions of TikTok as an entertainment-first platform [9].

Methodological considerations for short-form video metrics

Evaluating medical education on algorithmic platforms requires careful metric framing. Traditional engagement rates assume a bounded ratio where interactions occur primarily within a known follower base. On discovery-driven feeds, non-follower impressions balloon the numerator relative to a small follower denominator. Developing standardized, video-native quality and engagement frameworks-such as per-view interaction ratios or watch-time retention-will be essential for future digital health communication research [10].

Limitations

This study has several limitations. First, data collection represents a single cross-sectional snapshot; interaction metrics fluctuate rapidly over time. Second, restricting inclusion to US-based, English-speaking ID physicians and pharmacists excluded international creators and allied health professionals [9]. Third, third-party analytics platforms such as Collabstr may exhibit minor reporting variations based on data refresh cycles [8]. Fourth, the final sample size (n=13) limits generalizability across broader medical specialties. Fifth, our central comparison between cohort interaction-to-follower ratios and established industry engagement benchmarks (1.0%-5.0%) is limited by differing underlying denominators [8]. Because TikTok's recommendation feed exposes videos heavily to non-followers, ratios calculated against creator follower counts are subject to discovery-driven inflation. Consequently, high ratios reflect algorithmic content distribution and interaction efficiency rather than a direct mathematical equivalence to standard follower-bound engagement metrics. Finally, using broad consumer hashtags such as #antibiotics to maximize search sensitivity introduced initial selection noise, requiring extensive manual screening to filter non-specialist and lay content.

## Conclusions

Credentialed ID physicians and pharmacists remain starkly underrepresented on TikTok relative to their established presence on traditional microblogging platforms. Despite this pronounced scarcity, active clinician-creators demonstrate remarkable interaction efficiency, consistently generating audience engagement metrics that exceed standard digital healthcare benchmarks. This hyper-engagement is particularly pronounced among smaller, nano-tier accounts, underscoring how TikTok's recommendation algorithm privileges topical content over pre-existing follower counts. Consequently, short-form vertical video represents an untapped, high-leverage channel for medical specialists to combat widespread health misinformation and disseminate evidence-based public health messaging. To capitalize on this algorithmic reach, academic medical centers and public health institutions must actively incentivize clinician-led content creation by offering formal training and production support. Furthermore, modernizing academic promotion frameworks to recognize digital scholarship will be critical to encouraging sustained faculty participation in digital space. Ultimately, fostering a robust, verified expert presence on emerging media platforms is vital to protecting public health in an increasingly digital information ecosystem.
